# Avian influenza: genetic evolution under vaccination pressure

**DOI:** 10.1186/1743-422X-5-15

**Published:** 2008-01-24

**Authors:** Magdalena Escorcia, Lourdes Vázquez, Sara T Méndez, Andrea Rodríguez-Ropón, Eduardo Lucio, Gerardo M Nava

**Affiliations:** 1 Departamento de Producción Animal Aves. Facultad de Medicina Veterinaria y Zootecnia. Universidad Nacional Autónoma de México, D. F. 04510, México; 2 Unidad de Genética de la Nutrición. Instituto Nacional de Pediatría, D. F, 04530, México; 3 Investigación Aplicada, S. A. de C. V. 7 Norte 416, Tehuacán, Puebla, 75700, México; 4 Laboratory of Mucosal Biology. University of Illinois at Urbana-Champaign, 61801, USA

## Abstract

Antigenic drift of avian influenza viruses (AIVs) has been observed in chickens after extended vaccination program, similar to those observed with human influenza viruses. To evaluate the evolutionary properties of endemic AIV under high vaccination pressure (around 2 billion doses used in the last 12 years), we performed a pilot phylogenic analysis of the hemagglutinin (HA) gene of AIVs isolated from 1994 to 2006. This study demonstrates that Mexican low pathogenicity (LP) H5N2-AIVs are constantly undergoing genetic drifts. Recent AIV isolates (2002–2006) show significant molecular drifts when compared with the H5N2 vaccine-strain or other field isolates (1994–2000). This study also demonstrates that molecular drifts in the HA gene lineages follow a yearly trend, suggesting gradually cumulative sequence mutations. These findings might explain the increasing incidence of LP H5N2 AIV isolated from commercial avian farms. These findings support recent concerns about the challenge of AIV antigenic drift and influenza epidemics.

## Findings

Avian influenza virus (AIV) is a member of the *Orthomyxoviridae *family, *Influenzavirus A *genus. AIV is characterized by its ability to undergo constant antigenic changes [1]. AIV envelope contains two major glycoproteins, hemagglutinin (HA) and neuraminidase (NA) [2]. The HA/NA proteins play a key role during cellular infection. Different HA/NA combinations allow AIV subtype discrimination. Depending on the damage caused to avian species, AIVs are categorized as either high or low pathogenicity (HP and LP, respectively).

In Mexico, LP AIV was first detected in May 1994, among commercial farms. Since then, an Avian Influenza National Campaign established by the Mexican Ministry of Agriculture, Livestock, Rural Development, Fisheries and Food (SAGARPA, its Spanish acronym), is in operation. The purpose of this campaign is to eradicate the subtype H5N2 LP AIV that is still present in specific areas of Mexico. Vaccination of commercial flocks is one of many strategies for this campaign. The vaccine strain officially authorized, as seed for commercial vaccine production is the A/Ck/México/CPA-232/94 (H5N2), isolated in 1994 [3].

The use of the commercial vaccine in Mexico was originally aimed to eradicate HP AIV and this was accomplished in June 1994. Then, the decision was made to continue vaccinating in order to protect commercial flocks from LP AIV outbreaks. Nevertheless, 13 years after the use of the vaccination started and more than 2 billion doses were used in the Mexican avian industry [4], an increase in respiratory signs of disease has been observed in vaccinated, field challenged birds.

The current increase in incidence of AIV infection is most likely related to antigenic drifts occurred in field AIV strains [5]. Moreover, veterinary services revealed more than two log differences in cross hemagglutination inhibition tests between field isolates and the vaccine seed virus (Lucio E., unpublished).

Vaccination programs produce faster antigenic drifts of human and avian influenza viruses [6]. Nevertheless, there are few biological systems to explore the dynamic of influenza virus evolution. In the present study, we explored the use of the Mexican aviculture as an example of a suitable model to evaluate the evolutionary properties of endemic AIV under high vaccination pressure. The Mexican aviculture system offers an excellent model to study AIV genetic evolution under high vaccination pressure for two important grounds: i) avian influenza vaccination is a regular veterinary practice, and ii) poultry systems are characterized by high avian population density per production unit. We compared HA gene sequences from AIVs isolated between 1994 and 2000 [3], more recent isolates (2002 to 2006) from vaccinated birds showing clinical manifestations of avian influenza, and the A/Chicken/Hidalgo/232/94 vaccine strain.

For the present study, we used the complete collection of eighteen AIV strains isolated from years 2002 to 2006 from nine different regions of Mexico. These strains were isolated and guarded by an officially certified laboratory to issue reports for control and eradication of avian influenza in Mexico. All AIVs were obtained from vaccinated birds showing clinical signs of avian influenza. Viral RNA extraction from allantoic fluid was performed using conventional methods [7]. Reverse transcriptase PCR (RT-PCR) was used for the amplification of the HA cleavage site sequence, a marker for the virulence potential of avian influenza viruses [8]. Sequencing of the HA gene segments was performed using the 3730Xl automated sequencer (Applied Biosystems, CA. USA). The nucleotide sequences obtained in this study (available from the authors upon request) and sequences retrieved from the GenBank database under accession numbers [GenBank:AY497063 to AY497096] produced from Mexican AIVs [3] were analyzed by using the CLUSTALW package and then edited using the jalview program [9]. Because the sequences retrieved from GenBank differed in extension, the flanking N-term and C-term regions were removed for the alignment. Edited sequences were re-aligned using the ClustalX 1.81 program [10] with the following parameters. Pair-wise: gap opening = 10.0, gap extension = 0.10; Alignment: gap opening = 10.0, gap extension = 0.2, and Gonnet series weight matrix. Phylogenetic trees were constructed with the Maximum Parsimony method [11] using the PAUP* 4.0 b10 program [12]. Trees were rooted using [GenBank:AY497063] (vaccine strain) as ancestral nucleotide sequence. The statistical significance of branch order was estimated by the generation of 1000 replications of bootstrap re-sampling of the originally-aligned nucleotide sequences.

**Figure 1:**
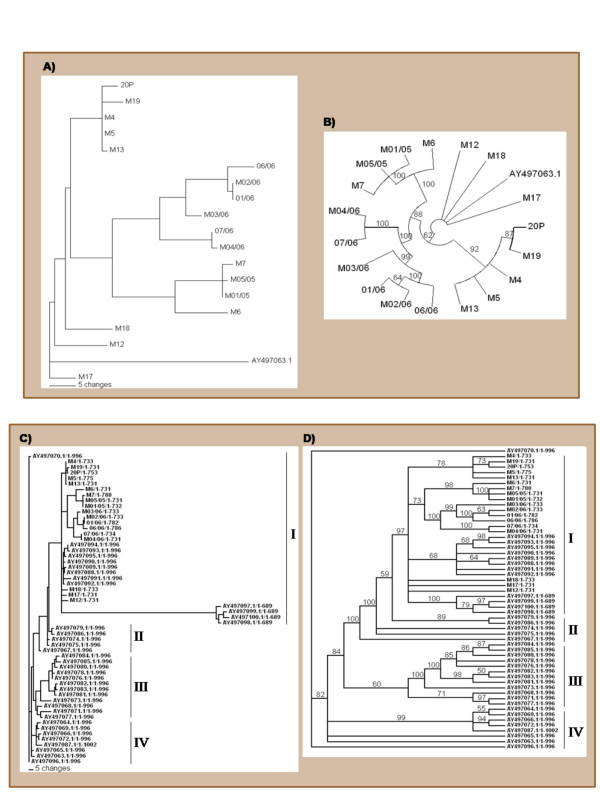
**Current Mexican avian influenza virus lineages**. Phylogenetic rooted trees based on the nucleotide sequence of the hemagglutining (HA) gene (cleavage site sequence) from avian influenza virus (AIV) strains. Trees were constructed using maximum parsimony and best heuristic tree search analysis. The analysis shows the relationships of nucleotide sequences of the HA gene. The [GenBank:AY497063.1] nucleotide sequence was used for rooting. A) Phylogram tree and B) Circular tree showing bootstrap values (numbers on braches) after 1,000 replicates. Parts A and B include recent AIV isolates from years 2002 to 2006. Sequence labels are contractions of the official identifications: *sequence label (official code) *M4 (A/Chicken/Querétaro/M4/02), M5 (A/Chicken/Hidalgo/M5/02), M12 (A/Chicken/Nuevo León/M12/02), M13 (A/Chicken/San Luis Potosí/M13/02), M17 (A/Chicken/Puebla/M17//02), M18 9 (A/Chicken/San Luis Potosí/M18//02), M19 (no official ID), 20P (no official ID), M6 (A/Chicken/Colima/M6/05), M7 (A/Chicken/Jalisco/M7/05), M01/05 (A/Chicken/Aguascalientes/01-05/05), M05/05 (A/Chicken/Puebla/05-05/05), 01/06-A/Chicken/Puebla/01-06/06, M02/06 (A/Chicken/Estado de México/02-06/06), M03/06 (A/Chicken/Estado de México/03-06/06), M04/06 (A/Chicken/Puebla/04-06/06), 06/06 (A/Chicken/Puebla/06-06/06), 07/06 (A/Chicken/Puebla/07-06/06). For each official code: serotype/host/location/reference/year of isolation.C) Phylogram tree and D) Rectangular tree showing bootstrap values (numbers on braches) after 1000 replicates. Parts C and D include AIV isolated from 1994 to 2006. Current Mexican AIV lineages are indicated by I to IV. Sequence labels correspond to GenBank accession numbers/nucleotide fragment size (HA gene cleavage site) used for phylogenetic analysis.

For the eighteen HA sequences from recent (2002–2006) Mexican AIV isolates, the nucleotide sequences similarity varied between 99.6 and 86.8%. Phylogenetic trees show that these HA gene sequences are grouped into 4 phylogenetic lineages that in general, matched the years of isolation (Figure 1A–B). For the construction of phylogenetic trees, the vaccine strain was used as the ancestral sequence (phylogenetic root) in order to determine the genetic variation between field isolates and the vaccine strain. Three of the 2002 sequences were the most phylogenetically closely related to the vaccine strain. Same results were obtained when unrooted phylogenetic trees (not shown) were constructed. These results indicate that the HA gene from field strains constantly experience genetic variation, and the accumulation of these genetic drifts allows distinguishing genetic changes that can be related to the year of isolation.

Conversely, the construction of phylogenetic trees with the HA sequences from 1994–2002 isolates and from 2002–2006 isolates (present study) show that the HA sequences are grouped in four (I-IV) lineages. Likewise, these 4 viral Mexican AIV lineages follow an isolation year trend (Figures 1C–D). Same lineages were obtained when unrooted phylogenetic trees were constructed (not shown). These four lineages include sequences previously classified as Jalisco, A, and B [3]. Since 2004, no other additional studies have evaluated the evolution of LP AIVs circulating in Mexico. In here, we provide evidence of the currently existing Mexican AIV lineages.

Mexico has been world's first country in adopting vaccination of commercial poultry as one of several measures for avian influenza control and eradication. Since 1995, more than 2 billion vaccine doses have been used in the Mexican avian industry [4].

Results from the present study show that recent Mexican LP H5N2 AIV field isolates show constant drifts in their genetic information. The phylogenetic analysis shows that all eighteen recent isolates (2002–2006) have undergone important drifts in the HA gene sequence as compared with vaccine strain. This finding indicates that it is important to consider new prophylactic strategies against avian influenza in Mexico as well as in other countries which are currently using the same vaccine. Swayne *et al*. (2000) reported that the percent homology of the HA gene sequence between field isolates and the vaccine strain is essential to decrease spread levels of field AIV strains [13]. The genomic variation found in the present study might explain the permanence of LP AIV in Mexico since 1994, despite the use of both vaccination and stringent biosecurity measures [13].

In previous reports, homology in HA sequences of AIVs isolated between 1994–2002 was 86.0–99.8% [3]. Comparable homology values (86.8–99.0%) were estimated in the present study within the 2002–2006 AIV isolates. Sequences from recent (2002–2006) AIV isolates and the vaccine strain show significant phylogenetic divergence. This could explain the clinical signs observed in the vaccinated chickens.

It is possible that avian influenza vaccination has resulted in accelerated genetic drifts in the HA gene sequence, as previously suggested by Lee *et al*. (2004). However, Ellis and Zambon (2001), Gambaryan *et al*. (2006), Widjaja *et al*. (2006) showed that viral replication *per se *allows for the expression of drifts among subsequent viral populations [14-16].

The AIVs sequences obtained in the present study are closely related to lineage B reported by Lee *et al*. (2004), which are viruses phylogenetically more distant from the vaccine strain [3]. Therefore, it is important to implement more rigorous requisites than those proposed by the World Organization of Animal Health (OIE) which only considered protection afforded by the vaccine in terms of bird survival rates. Together with other measures, protection in terms of reduced viral spread from vaccine strains has also been an indispensable requirement to achieve the goal of eradication.

The present phylogenetic analysis based on the analysis of HA gene shows that cumulative genetic drifts in the HA gene allow distinction between new and old AIV lineages. The occurrence of influenza outbreaks could be associated to antigenic drifts, in which changes in viral antigens provide and evolutionary advantage to re-infect the same host [17]. All these together, support the recent concerns about the challenge of AIV antigenic drift and influenza epidemics.

Finally, we consider that the current biological system (Mexican avian industry) is a suitable model to evaluate the evolutionary properties of endemic AIV under high vaccination pressure. Further studies need to be conducted to compare HA sequences from countries without avian influenza vaccine.

## Abbreviations

AIV: Avian influenza virus; HA: Hemagglutinin; HP: High pathogenicity; LP: Low pathogenicity; NA: Neuraminidase; SAGARPA: Mexican Ministry of Agriculture, Livestock Rural Development, Fisheries and Food; PAUP*: Phylogenetic Analysis Using Parsimony; RNA: Ribonucleic acid; RT-PCR: Reverse transcriptase polymerase chain reaction

## Competing interests

The author(s) declare that they have no competing interests.

## Authors' contributions

ME, EL and GMN designed research; ME, LV, and ARR performed research; EL and STM contributed reagents and analytic tools; GMN and ME analyzed data; GMN and ME wrote the paper. All coauthors read and approved the final manuscript.
